# A critical analysis of the cycles of physical activity policy in England

**DOI:** 10.1186/s12966-015-0169-5

**Published:** 2015-02-01

**Authors:** Karen Milton, Adrian Bauman

**Affiliations:** Research Fellow, School of Public Health, Level 6 Charles Perkins Centre, University of Sydney, Building D17, Sydney, NSW 2006 Australia; Sesquicentenary Professor of Public Health, School of Public Health, Level 6 Charles Perkins Centre, University of Sydney, Building D17, Sydney, NSW 2006 Australia

**Keywords:** Physical activity, Policy, Recommendations, Surveillance, Public education

## Abstract

**Background:**

There has been increasing focus on the importance of national policy to address population levels of physical inactivity. Components of a comprehensive national physical activity policy framework include: 1) national recommendations on physical activity levels; 2) setting population goals and targets; 3) surveillance or health monitoring systems; and 4) public education. The aim of the current paper was to analyse the policy actions which have addressed each of these elements in England and to identify areas of progress and remaining challenges.

**Methods:**

A literature search was undertaken to identify past and present documents relevant to physical activity policy in England. Each document was analysed to identify content relevant to the four key elements of policy which formed the focus of the current research.

**Results:**

Physical activity recommendations are an area where England has demonstrated a robust scientific approach and good practice; however, the physical activity campaigns in England have not been sufficiently sustained to achieve changes in social norms and behaviour. The setting of physical activity targets has been unrealistic and continuous changes to national surveillance measures have presented challenges for monitoring trends over time.

**Conclusions:**

Overall, physical activity policy in England has fluctuated over the past two decades. The variations and cycles in policy reflect some of the challenges in implementing and sustaining physical activity policy in the face of political changes, changes in government direction, and changing opportunities to profile active lifestyles.

## Background

Non-communicable diseases (NCDs) are the leading cause of global mortality, accounting for more premature deaths each year than all other causes combined [1]. Physical inactivity is one of the four key behavioural risk factors for NCDs [2]; yet recent surveillance data suggests that over 40% of adults in England do not meet recommended physical activity levels [3]. As a result, physical inactivity is thought to cause 3.1% of morbidity and mortality in England, and is responsible for 35,000 deaths annually [4].

The World Health Organization’s (WHO) Global Strategy on Diet, Physical Activity and Health highlighted the importance of scaling up national policy action to address physical activity [5]. The development of a national policy framework is recognised as an important step in raising the profile of physical activity as a priority area and providing a coherent action plan or programme of activities aimed at increasing the population prevalence of physical activity [6]. The need to scale-up policy level interventions for physical activity has recently been reinforced at the highest levels, through both the United Nations Declaration [7] and the WHO Global NCD Action Plan 2013–2020 [8].

A report published by the WHO Regional Office for Europe in 2011 identified 17 key elements of a national physical activity policy, which are summarized below [9,10]. These elements relate to the process of policy development, the content of policy, and its implementation. The focus of the current paper was on the content of national policy. Four key areas of policy content, which are often considered the ‘cornerstones’ of a successful national policy framework are: 1) national recommendations on physical activity levels; 2) national goals and targets; 3) surveillance or health monitoring systems; and 4) public education [11,12]. The aim of the current paper was to analyse the past and present policy actions which address each of these policy components in England and to identify areas of progress and remaining challenges. The government in England has recognised the links between physical inactivity and disease since the mid-1970s [13,14], however it was not until the early-1990s that physical activity promotion began to be considered as a government responsibility. Therefore the early-1990s to the early-2010s formed the focus of the current review, reflecting a twenty year history.

### The 17 elements identified as important for a successful national approach to physical activity promotion

1. Consultative approach in development
2. Evidence based
3. Integration across other sectors and policies
4. National recommendations on physical activity levels
5. National goals and targets
6. Implementation plan with a specified timeframe for implementation
7. Multiple strategies
8. Evaluation
9. Surveillance or health monitoring systems
10. Political commitment
11. On-going funding
12. Leadership and coordination
13. Working in partnership
14. Links between policy and practice
15. Communication strategy
16. Identity (branding/logo/slogan)
17. Network supporting professionals

## Methods

A literature and web-search was undertaken to identify key documents related to physical activity policy in England. The web-search mainly focused on the websites of the Department of Health (DH) and the Department for Culture Media and Sport (DCMS), as these departments are largely responsible for the promotion of physical activity and sport in England. We used the search term ‘physical activity’ and all identified documents were considered. This search identified many key policy documents for physical activity, including those related to physical activity recommendations (‘At Least Five a Week’ and ‘Start Active Stay Active’), and key strategy documents (including Be Active Be Healthy, Game Plan, and the Legacy Action Plan). Other websites which were searched included Sport England, the DCMS funded body responsible for the delivery of sport in England, and the Health and Social Care Information Centre, the body responsible for conducting the national surveillance system, the Health Survey for England. The reference lists of all identified documents were screened to identify other relevant documents, which were subsequently obtained. The reference lists of these documents were also screened in an iterative process, until no further documents could be identified.

The web-search was supplemented by a search of the scientific literature. A range of search terms were entered into the PubMed database including: “physical activity recommendations”, “physical activity surveillance”, “physical activity trends”, “Health Survey for England”, “Active People Survey”, “physical activity campaign”, “Active for Life” and “Change4Life”.

Each document was screened to identify content relevant to the four key elements of policy which formed the focus of the current research. Any documents which did not contain content related to at least one of the four areas were excluded from the analysis. For all documents which did contain relevant content, a more in-depth analysis was undertaken to examine the substance and meaning of the documents by taking into account both the text and its specific context.

## Results

This review focused on four key aspects of physical activity policy: 1) national recommendations on physical activity levels; 2) national goals and targets; 3) surveillance or health monitoring systems; and 4) public education. Developments in relation to each theme area are presented below, which is followed by a summary of key documents and milestones.

### National recommendations on physical activity levels

The development of physical activity recommendations is important for providing consensus on the amount of physical activity needed to promote health and reduce the risk of NCDs. Physical activity recommendations can facilitate clear communication to the public about the benefits of physical activity and can provide a foundation for national surveillance and target setting.

In the 1990s in England, the Health Education Authority (HEA) was responsible for advising the government on health related strategy, including physical activity. In April 1994, the HEA held a symposium aimed at reaching agreement on the amount of physical activity to be recommended for health [15]. Over 40 national and international experts spent three days reviewing the evidence and reaching consensus on what messages should be promoted [15]. This symposium informed the Department of Health’s 1996 *Strategy Statement on Physical Activity* [16]*.* This document recommended ‘30 minutes of moderate intensity physical activity on at least five days of the week’ and for those already doing some vigorous physical activity ‘three periods per week of vigorous intensity physical activity of 20 minutes each’ [16]. These physical activity guidelines were endorsed nearly a decade later (in 2004) in a landmark document from the Chief Medical Officer, entitled *At Least Five a Week* [17]*.* The physical activity guidelines for England were similar (although not identical) to those published by the American College of Sport Medicine (ACSM) and the Centres for Disease Control and Prevention (CDC) in the United States of America (USA) [18], which received government endorsment in *Physical Activity and Health: A Report of the Surgeon General*, in 1996 [19].

Only four years later (in 2008), the Department of Health in England commissioned a review and update of the physical activity recommendations. This review was prompted by new evidence on the relationship between physical activity and health, updates to the physical activity recommendations in other countries (e.g. USA and Canada, as well as the ongoing WHO process to develop Global recommendations), and differences between the existing guidelines in England, Wales, Scotland and Northern Ireland. The review process involved a wide-consultative approach, including reviews of the evidence undertaken by expert advisory groups, a two-day scientific meeting, and a national web-based consultation [20]. The outcome of this review was published three years later as *Start Active, Stay Active: A Report on Physical Activity from the Four Home Countries Chief Medical Officers* [21], which announced for the first time, a common set of recommendations across the United Kingdom (UK). This document provided updated guidelines for children, young people and adults, and included new guidelines for early years (under 5 years) and older adults (65+ years). The new recommendations stated that “*Adults should aim to be active daily. Over a week, activity should add up to at least 150 minutes (2½ hours) of moderate-intensity activity in bouts of 10 minutes or more – one way to approach this is to do 30 minutes on at least 5 days a week. Alternatively, comparable benefits can be achieved through 75 minutes of vigorous-intensity activity spread across the week or a combination of moderate and vigorous intensity activity”* [21]*.* The new recommendations also highlighted the potential risks of sedentary behaviour for all age groups.

Despite the effort invested into the review process and the development of the new UK guidelines, limited effort was invested in their dissemination and communication. The Department of Health simply announced that it would work with the Change4Life and NHS Choices teams to ensure that the guidelines were being disseminated to the public via “*various campaigns*” [22]. Thus, there was no clear translation of the guidelines into appropriate ‘messages’ to be disseminated to the public. As a result, two years after their launch it was reported that over 80% of the public were unable to accurately recall the current guidelines [23].

### National goals and targets

Identifying specific and measurable targets within national policy can help to ensure that clear policy actions are identified and implemented and that relevant agencies are held accountable for progress. Although many policy documents emphasise the importance of ‘increasing’ physical activity, the clear definition of specific and measureable targets is less common.

#### Game plan

In England, the first national policy document to specify quantifiable targets for physical activity prevalence was *Game Plan*, published by the DCMS Strategy Unit in 2002 [24]. Although *Game Plan* was published by the sports sector, it contained many references to broader physical activity, with a stated aim of encouraging a *“mass participation culture (with as much emphasis on physical activity as competitive sport)”* [24]*.*

This document stated a target that 70% of the population would undertake recommended physical activity levels by 2020. At the time *Game Plan* was published, just 32% of the population attained the recommended levels of physical activity. Therefore achievement of this target would necessitate doubling participation in physical activity, which would require an annual increase of 2%. Not only was *Game Plan* extremely ambitious in its target to move from 32% to 70% of the population meeting recommended physical activity levels, it also failed to identify any clear strategy or investment for how this target would be achieved.

The *Game Plan* target was inspired by Finland, where at the time an estimated 70% of the population were reaching the recommended 30 minutes of moderate activity on 5 days of the week [24]. Finland has a long and sustained investment in promoting physical activity and is one of the few countries (along with Canada) which has demonstrated long-term increasing trends in leisure time physical activity [25,26]. Even Finland, however, has not achieved an annual increase in physical activity prevalence of 1%, let alone 2% - the amount required to achieve the *Game Plan* target.

#### Legacy action plan

On 6^th^ July 2005 it was announced that London would host the 2012 Olympic and Paralympic Games. This provided a catalyst for national policy focused on how the Olympics could be used as a platform for the promotion of sport and physical activity. In 2008, DCMS released *Before, During and After: Making the Most of the London 2012 Games* [27]. Informally known as the ‘Legacy Action Plan’, this document set out the government’s intention to make the UK a world-leading sporting nation, with the ambition of achieving 4^th^ position on the medal tally at the 2012 Olympic Games and at least 2^nd^ position on the medal tally at the 2012 Paralympic Games. However, both the 2012 Games bid and the subsequent *Legacy Action Plan* used the rhetoric of wider participation in physical activity, with the aim of getting two million more people ‘active’ by 2012. ‘Active’ was defined as achieving a minimum of three 30 minute sessions of at least moderate intensity activity per week (quite inconsistent with all previous Department of Health physical activity recommendations, which had focused on 30 minutes on five or more days of the week).

Prior to the publication of the *Legacy Action Plan*, a cross-government review (unpublished) concluded that Sport England should take responsibility for sport and that the Department of Health should take responsibility for wider physical activity promotion (personal communication with the Department of Health). Therefore, whereas *Game Plan* had addressed sport and physical activity collectively, with cross-department targets and actions, the promotion of sport and physical activity were now considered distinct from one another, requiring different approaches and separate agencies to take leadership. The overarching aim of getting two million more people active was thus split between Sport England getting one million more people active through sport and the Department of Health getting one million more people active through participation in a broader range of physical activities [28]. Achievement of the target would be measured using Sport England’s Active People Survey [27].

However, between the publication of the *Legacy Action Plan* (June 2008) and the 2012 London Olympic and Paralympic Games there was a general election and a change in government. When a new government came into power in May 2010, the Department of Health’s one million target was *“quietly dropped”* [29]. Subsequently in 2011, following *“negligible progress”* the Sport England target to get one million more people active through sport was also abolished [30].

### Surveillance or health monitoring systems

The establishment of a sustained national surveillance system is necessary for monitoring trends in population prevalence of physical activity over time. The first national survey on physical activity in England was coordinated by the HEA and undertaken between 1990 and 1991. This ‘Allied Dunbar National Fitness Survey’ focused on the measurement of physical activity patterns and fitness among the adult population in England [31]. The results showed that 70% of the adult population were insufficiently active to benefit their health. The Allied Dunbar Survey was never repeated, but around the same time (1991) the Department of Health established the Health Survey for England. More recently, another national surveillance system, the Active People Survey, has been established.

#### Health survey for England

The Health Survey for England was established to provide ongoing population surveillance data on various aspects of health including eating habits, smoking, and physical activity. The annual interview-based survey used representative population samples ranging from 3,000 adults (1991) to 15,000 adults (2008). Physical activity was included annually for the first four years of the survey (1991 to 1994), but since then has been included intermittently, in 1997–1999, 2002–2004, 2006–2008 and in 2012 [3].

A key element of a national surveillance system is that the questions remain exactly consistent, allowing for monitoring of trends over time [32,33]. However, the physical activity questions in the Health Survey for England have been modified several times. From the original questions in the Allied Dunbar Survey, *“minor changes”* were made in 1992 and 1994, while *“more substantial revisions”* were made in 1997 and 1998 [3]. In 1999 a shorter version of the questions was produced and this survey was repeated in the 2002, 2003 and 2004 surveys. The 2006 survey reverted back to a *“slightly modified”* version of the long questionnaire [3]. An *“enhanced”* version of the questionnaire was developed for the 2008 survey, and the survey was modified again for 2012 [3]. Further details of the changes which have been made to the Health Survey for England are reported elsewhere [3,34]. These changes to the wording of the questions have masked trends in physical activity over time, due to an inability to differentiate true changes in physical activity from differences in the way physical activity was reported or computed due to changes in the questions [34].

Between 1999 and 2004 (when the physical activity questions remained unchanged), data from the Health Survey for England showed that the prevalence of adults meeting recommended physical activity levels increased from 46.8% in 1999 to 48.5% in 2004 [34]. In 2008, using different physical activity questions, the proportion of adults meeting recommended physical activity levels suddenly dropped to 34% [35].

The 2008 Health Survey for England included the collection of objective accelerometer data among a sub-sample of participants. Self-report data from this survey indicated that 39% of men and 29% of women aged 16 and over met the government’s recommendation for physical activity. However, accelerometer data showed that just 6% of men and 4% of women met recommended physical activity levels [35]. Although objective data collection methods are thought to provide a more accurate assessment of physical activity, the large discrepancies between the self-report and objective data caused confusion among the community, the media, and policymakers regarding prevalence and trends in physical activity in England.

For the 2012 survey the way in which the Health Survey for England data were analysed was modified to align with the new physical activity recommendations. Data from this survey indicated that 59% of adults were meeting recommended physical activity levels [3]. This estimate suggested a marked increase in physical activity from 2008, but the data were analysed in different ways. To resolve this issue, data from the 2008 survey were re-analysed, applying the “150 minute” recommendation. The results showed no change in physical activity prevalence over time, with a total of 59% of adults meeting recommended physical activity levels in both 2008 and 2012 [3]. This highlights that quite different conclusions can be reached from the same data, and reinforces the need for analytic standardisation.

#### Active people survey

Between October 2005 and October 2006 another population survey, the Active People Survey, was launched by Sport England. This was the largest sport and recreation survey ever conducted in England, which allowed for detailed sub-region analysis. The sample size of the first survey exceeded 360,000 adults (≥16 years), but at substantial costs of over £5,500,000 [36]. Subsequent surveys have been conducted with sample sizes between 160,000 and 195,000 participants, at a cost of approximately £2.5 to £3 million [36].

The Active People Survey was not conducted in 2006–2007, but since 2007–2008 has been undertaken annually, and is conducted via telephone administered interviews. The Active People Survey originally asked about sport and recreation only and was focused on participation in sport, club membership, coaching, volunteering in sport, and the provision of sport opportunities within the community [37]. In 2009, the scope of the Active People Survey was broadened to include dance and active conservation/ gardening [37]. In 2012 the survey was extended further to include ‘active transport’ (i.e. walking and cycling) [37]. Therefore several minor and major changes have been made to the tool in its short history. These changes, combined with the varying ways in which the data have been analysed and reported, make it challenging to use the Active People Survey to ascertain trends in physical activity over time.

Furthermore, from 2012 the Active People Survey took over from the Health Survey for England as the primary data source to monitor the population prevalence of physical activity. Consequently, several questions on the Active People Survey were amended to align with the new physical activity recommendations. For example, whereas the Active People Survey previously captured activity undertaken in 30 minute bouts, for the 2012 survey this was reduced to ten minute bouts. Therefore, it was not possible to compare data from the 2012 survey with previous Active People Survey results. In addition it was no longer possible to assess progress against the government’s *Legacy Action Plan* target of getting two million more adults active.

It is not clear what has motivated the frequent changes to the national surveillance systems, but potential reasons include a desire to constantly improve the measures used or attempts to meet the needs of a broad range of stakeholders. Regardless of the reason, it is questionable whether the government in England has a genuine interest in monitoring sustained trends in physical activity prevalence – while frequent changes to the surveys have not allowed a robust assessment of progress, these changes may also be helping to mask the lack of progress in increasing physical activity levels in England.

### Public education

Public education, through large scale communication campaigns, is considered a cornerstone of physical activity promotion [8,38-40]. Large scale communication campaigns typically use a variety of mass media to convey key messages about the importance of being physically active, with the aim of influencing understanding, attitudes and physical activity behaviour. Over the past 20 years in England there have been two large scale communication campaigns, *ACTIVE for LIFE* and *Change4Life*.

#### ACTIVE for LIFE

In 1996 the Department of Health published a *Strategy Statement on Physical Activity* which set out three broad objectives around promoting key physical activity messages [16]. The main aim of the strategy was to increase public awareness of the health benefits of being active and to encourage adults to undertake at least five sessions of 30 minutes of moderate intensity activity per week. The main communication mechanism for these messages was the *ACTIVE for LIFE* campaign which was commissioned by the Department of Health to run from 1996 to 1998. Key elements of the campaign included posters, leaflets, postcards, two websites, and paid TV advertising [41]. In addition the campaign was supported by an extensive programme of public relations activities [41].

An evaluation of the campaign reported that it achieved acceptable coverage when compared with other physical activity campaigns and there was a small but significant increase in the proportion of people who were aware of the physical activity recommendations following the main television advertising element of the campaign [41]. However, the evaluation concluded that there was *“no evidence that ACTIVE for LIFE improved physical activity, either overall or in any subgroup”* [41]. The authors of this evaluation suggested that these findings *“point to the need to be realistic about the time that it takes to affect ingrained social trends”* [41].

#### Change4Life

Change4Life was the social marketing campaign element of the *Healthy Weight Healthy Lives* cross-government obesity strategy, published in January 2008 [42]. The national Change4Life campaign was launched in January 2009 and was initially set to run for a three-year period, with an investment of £75 million from the Department of Health. The campaign focused on encouraging people to *“Eat well, Move more, [and] Live longer”* and utilised a range of communication channels including paid television commercials, newspaper advertising, posters, a website, e-mail marketing, and educational resources.

According to a Department of Health report, the first year of Change4Life was extremely successful; in its first year, Change4Life successfully met and exceeded all of its targets – exceeding its target for logo recognition by 50% [43]. Over 400,000 families joined Change4Life in its first year and over 1 million mothers claimed to have made changes to their children’s behaviours as a direct result of the campaign [44]. In contrast however, data from the Health Survey for England showed that during the time of the Change4Life campaign, the proportion of children aged 5–15 years meeting recommended physical activity levels decreased. Data from the 2008 survey showed that 28% of boys and 19% of girls met recommended physical activity levels. However, in 2012 these values had dropped to 21% and 16% respectively [3].

In July 2010, following the change in government in England, it was announced that funding for the Change4Life campaign would be withdrawn. Health Secretary Andrew Lansley proclaimed that *"There has been a change of government and there will now be a change of approach. We will be progressively scaling back the amount of taxpayers' money spent on Change4Life and asking others, including the charities, the commercial sector and local authorities, to fill the gap."* [45]. The government has re-instated a relatively small financial contribution to the Change4Life campaign, and in 2013 a budget of £10.9 million was indicated [46]. The remainder of funding for the campaign now comes from a range of organisations, some of which produce the ‘harmful’ foods that Change4Life should be seeking to reduce. For example, commercial partners for the campaign currently include Mars, one of the world’s leading chocolate and candy manufacturers, and Britvic, a major manufacturer and distributor of soft drinks including Pepsi, Tango and 7-Up [47].

### Summary of key policy milestones

- **National recommendations on physical activity levels**
  - Strategy Statement on Physical Activity, 1996 [16]
  - At Least Five a Week, 2004 [17]
  - Start Active Stay Active, 2011 [21]
- **National goals and targets**
  - Game Plan, 2002 [24]
  - Before, During and After: Making the Most of the London 2012 Games, 2008 [27]
- **Surveillance or health monitoring systems**
  - Allied Dunbar National Fitness Survey (1991)
  - Health Survey for England (1991)
  - Active People Survey (2005)
- **Public education**
  - ‘ACTIVE for LIFE’ (1996)
  - ‘Change4Life’ (2009)

## Discussion

This paper critically reviews developments in four areas of physical activity policy in England: national recommendations on physical activity levels; national goals and targets; surveillance or health monitoring systems; and public education. Each of these elements contributes to a comprehensive national approach to physical activity promotion [10,48].

National recommendations on physical activity levels provide guidance on the frequency, duration, intensity, and type of physical activity needed to promote health and prevent disease. These recommendations are informed by epidemiological reviews of evidence on the dose–response relationship between physical activity and health. Physical activity recommendations are an area of policy where England has demonstrated a robust scientific approach and good practice. However, it is critical that physical activity recommendations are translated into public ‘messages’ and widely communicated. Indeed, the promotion of public awareness about physical activity has been identified as one of the ‘best buys’ in reducing population levels of physical inactivity [8].

Mass media campaigns are one vehicle for communicating physical activity messages [39]. In England, there have been two large scale physical activity campaigns, ACTIVE for LIFE in the mid-to-late 1990s and Change4Life more recently. Each was funded for a three-year period; however, best practice research suggests that campaigns need to be standardised, sequenced, and sustained over many years in order to achieve the sought-after changes in social norms and behaviour [49,50]. Thus there is a need to secure longer-term government commitment to these types of campaigns if they are to exert any influence. It has already been noted elsewhere, that *“it will take much longer than the brief public education campaigns in (…) the United Kingdom to achieve long-term influence on community understanding, message awareness and ‘brand’ recognition”* [49].

Previous research suggests that these types of educational campaigns can raise awareness, but in isolation are unlikely to lead to physical activity behaviour change [38,41,51]. It is essential that public education is supported by sustained and coordinated cross-sectoral investment, for example programmes and infrastructure to increase opportunities for people to be physically active.

A good example of delivering public education was the integrated ‘Push Play’ campaign in New Zealand. This was a nine year multi-component campaign, with culturally salient messaging, consistent branding, and a range of events and community-wide programmes implemented under the ‘Push Play’ banner [49]. Another example was the ParticipACTION campaign in Canada which ran for almost 30 years, with strong branding and a range of population group-specific initiatives to support physical activity behaviour change [39]. Research has shown that when delivered with sufficient reach, and well supported by physical activity opportunities, these community-wide initiatives can lead to significant increases in awareness of physical activity messages, intentions to be more active, and also physical activity behaviour [49].

It is also important to consider the role of corporate sponsorship in the delivery of physical activity campaigns and programmes. Although sponsorship provides useful revenue, target audiences transfer some *“image value”* from one party to another [52]. Thus when the sponsor has images that are dissimilar or inconsistent with health (such as sugar sweetened beverage manufacturers or fast food corporations) there is potential for these partnerships to have an adverse impact on the image of physical activity and health. Therefore, policymakers should align themselves with partners and sponsors who have shared public health goals and interests and a positive health-related ‘image’.

National policies often state intentions to ‘increase’ physical activity levels. More specific and measureable population targets can be used to evaluate the success or failure of a policy and provide a level of national and regional accountability. In England physical activity targets have been unrealistic, especially the *Game Plan* premise of increasing physical activity rates from 32% to 70% in 18 years. Change of this magnitude has never been observed anywhere in the world.

Some countries have been much more cautious (and realistic) than England in the establishment of measurable physical activity goals. In Switzerland for example, the Swiss Sport Policy included a target *“to stabilise and then increase by 1% per year the proportion of physically active people”* [53]. This target was supported by clear objectives and a plan for action, and national surveillance data show that the policy was largely effective in achieving the target [54]. National physical activity goals and targets should reflect what is realistically achievable in a defined timeframe. Targets are unlikely to ever be achieved without sufficient multi-program and cross-sectoral support to enable population change. Establishing short-term process implementation indicators as well as longer-term targets extending beyond single political cycles are essential if progress is to be assessed [55].

National surveillance systems need to be standardized and sustained to monitor physical activity and also to assess antecedent environments and programs indicated in national plans [33]. In England there have been continuous changes to the measures used to assess physical activity, changes to the agencies responsible for surveillance, and the development of two national and concurrent surveillance systems, both at huge costs. Despite a 20 year history of well-meaning investment in physical activity surveillance in England, repeated changes to the survey instruments has provided limited information on long-term trends in physical activity.

There are several good examples of sustained national physical activity surveillance, particularly in Finland and the USA. The annual surveys of the Public Health Institute in Finland have been undertaken since 1978 [56]. Despite including only one question on physical activity, the question has remained unchanged and thus has provided comparable data on physical activity trends in Finland for 35 years [56]. Similarly, the BRFSS in the USA, which also includes one question on physical activity, has remained mostly consistent over a 20 year period [57]. These data monitor trends, evaluate effectiveness of policy and programs, and underpin advocacy efforts for greater political support and investment.

Overall, physical activity policy in England has fluctuated over the past two decades. The four aspects of policy examined in the current review are essential to underpin a strong national policy framework. Yet despite a 20 year history of physical activity policy, the building blocks of national physical activity policy have still not been firmly established. The greatest progress has been made in national physical activity recommendations. However, the establishment of realistic national targets, a consistent and sustained national surveillance system, and a sustained public education campaign are areas requiring consistent policy implementation and maintenance in the long-term. The variations and cycles in policy reflect some of the challenges in implementing and sustaining physical activity policy in the face of political changes, changes in government direction, and changing opportunities to profile active lifestyles.

## Conclusions

There has been increasing focus on the importance of national policy to address population levels of physical inactivity, and over the past few years research has identified the key elements of a successful national physical activity policy framework [10-12]. This paper has identified areas of progress as well as remaining challenges in four key aspects of national physical activity policy in England. The findings highlight the importance of developing and implementing evidence based policy approaches, evaluating the effectiveness of policy, and sharing experiences (both successes and failures) to inform future policy development.
